# Effects of testosterone treatment on body fat and lean mass in obese men on a hypocaloric diet: a randomised controlled trial

**DOI:** 10.1186/s12916-016-0700-9

**Published:** 2016-10-07

**Authors:** Mark Ng Tang Fui, Luke A. Prendergast, Philippe Dupuis, Manjri Raval, Boyd J. Strauss, Jeffrey D. Zajac, Mathis Grossmann

**Affiliations:** 1 Department of Medicine Austin Health, University of Melbourne, 145 Studley Road, Heidelberg, VIC 3084 Australia; 2 Department of Endocrinology, Austin Health, 300 Waterdale Road, Heidelberg West, VIC 3081 Australia; 3 Department of Mathematics and Statistics, La Trobe University, Plenty Road & Kingsbury Drive, Melbourne, VIC 3086 Australia; 4 Department of Medicine, School of Clinical Sciences, Monash University, Clayton Road, Clayton, VIC 3800 Australia

**Keywords:** Testosterone, Obesity, Caloric restriction, Body composition

## Abstract

**Background:**

Whether testosterone treatment has benefits on body composition over and above caloric restriction in men is unknown. We hypothesised that testosterone treatment augments diet-induced loss of fat mass and prevents loss of muscle mass.

**Methods:**

We conducted a randomised double-blind, parallel, placebo controlled trial at a tertiary referral centre. A total of 100 obese men (body mass index ≥ 30 kg/m^2^) with a total testosterone level of or below 12 nmol/L and a median age of 53 years (interquartile range 47–60) receiving 10 weeks of a very low energy diet (VLED) followed by 46 weeks of weight maintenance were randomly assigned at baseline to 56 weeks of 10-weekly intramuscular testosterone undecanoate (*n* = 49, cases) or matching placebo (*n* = 51, controls). The main outcome measures were the between-group difference in fat and lean mass by dual-energy X-ray absorptiometry, and visceral fat area (computed tomography).

**Results:**

A total of 82 men completed the study. At study end, compared to controls, cases had greater reductions in fat mass, with a mean adjusted between-group difference (MAD) of –2.9 kg (–5.7 to –0.2; *P* = 0.04), and in visceral fat (MAD –2678 mm^2^; –5180 to –176; *P* = 0.04). Although both groups lost the same lean mass following VLED (cases –3.9 kg (–5.3 to –2.6); controls –4.8 kg (–6.2 to –3.5), *P* = 0.36), cases regained lean mass (3.3 kg (1.9 to 4.7), *P* < 0.001) during weight maintenance, in contrast to controls (0.8 kg (–0.7 to 2.3), *P* = 0.29) so that, at study end, cases had an attenuated reduction in lean mass compared to controls (MAD 3.4 kg (1.3 to 5.5), *P* = 0.002).

**Conclusions:**

While dieting men receiving placebo lost both fat and lean mass, the weight loss with testosterone treatment was almost exclusively due to loss of body fat.

**Trial registration:**

clinicaltrials.gov, identifier NCT01616732, registration date: June 8, 2012

**Electronic supplementary material:**

The online version of this article (doi:10.1186/s12916-016-0700-9) contains supplementary material, which is available to authorized users.

## Background

The obesity epidemic is associated with adverse health outcomes and high socioeconomic costs. Modest weight loss provides important health benefits, but successful weight loss is difficult to achieve and maintain. Although most studies testing interventions for obesity focus on body weight, excess body fat is considered responsible for most obesity-associated health risks and associated with increased mortality independent of body mass index (BMI) [1]. Adiposity contributes to loss of muscle mass and function, with sarcopenia increasing insulin resistance, a self-perpetuating phenotype termed “sarcopenic obesity” [2]. Therefore, the benefit of energy restriction may be limited by loss of lean body mass [2].

In men, obesity is the single most important factor associated with low testosterone, overriding the effects of age and comorbidities [3]. Obese men have 30 % lower total testosterone (TT) levels compared to lean men [3], and 40 % have levels lower than 12 nmol/L [3], the lower limit reported for healthy young men [4]. This reduction in total testosterone levels is in part due to the obesity-associated lowering in sex hormone binding globulin (SHBG). However, especially with more marked obesity, free testosterone levels are also reduced due to adiposity-associated suppression of the gonadal axis at the hypothalamic level. While the exact mechanisms are not fully understood, experimental studies in humans suggest that fat-derived adipokines and pro-inflammatory mediators may play a role in this central gonadal axis suppression [5]. In addition, preclinical evidence has shown that testosterone deficiency promotes adipose tissue accumulation but reduces myogenesis via an androgen receptor mediated pathway [6]. This bidirectional relationship between lowered testosterone and obesity is supported by clinical studies – weight loss increases testosterone proportionally to weight loss [7] and testosterone treatment reduces body fat [8].

Whether testosterone treatment augments fat loss additive to caloric restriction or prevents diet-associated loss of muscle mass is unknown. We conducted a randomised clinical trial in obese men with low to low-normal total testosterone to test the hypothesis that, following diet-induced loss of fat mass, testosterone treatment will prevent fat regain but maintain lean mass.

## Methods

### Study design

This 56-week, randomised, double-blind, placebo controlled trial (RCT) (ClinicalTrials.gov NCT01616732) was conducted at a tertiary referral centre (Austin Health, Melbourne, Australia). The study was approved by the Human Research Ethics Committee, Austin Health (HREC 2012/04495).

### Participants

Adult men aged 18–70 years recruited from the local community via print, radio and television advertisements were eligible to participate if they were obese (BMI ≥ 30 kg/m^2^) and had two TT levels of or below 12 nmol/L measured in the morning in the fasting state at least one week apart. Exclusion criteria were pathological androgen deficiency due to pituitary or testicular disease, testosterone treatment during the previous 12 months, prostate disease, cancer, haematocrit above 50 %, symptomatic ischaemic heart disease, cardiovascular event in preceding 12 months, congestive cardiac failure above NYHA Class I, blood pressure above 160/100 mm Hg despite antihypertensives, uncontrolled obstructive sleep apnoea, chronic kidney disease (estimated glomerular filtration rate < 40 mL/min), use of weight-altering medications including insulin and glucagon-like peptide 1-agonists, previous very low energy diet (VLED) failure, bariatric surgery, major depression, recreational drug use, and alcohol dependence. Each participant provided written informed consent prior to inclusion in the study.

### Randomisation and masking

Subjects were randomly assigned in a concealed 1:1 allocation to either testosterone or placebo using a block of size four with equal probability to the two treatments within four strata accounting for BMI (≤ or > 37 kg/m^2^) and age (≤ or > 60 years). The randomization sequence was generated by an independent statistician and implemented by the Austin Health clinical trials pharmacists. Participants, trial investigators and pharmacists were blinded to treatment allocation.

### Procedures

Men received either 1000 mg testosterone undecanoate (the standard ampoule strength in Australia) or visually identical placebo in oily base by deep intramuscular buttock injection at weeks 0 and 6 (manufacturer-recommended loading dose), and 10-weekly thereafter at weeks 16, 26, 36 and 46. The 10-weekly interval, in line with the manufacturer’s recommendations (10–14 weeks), was chosen to ensure therapeutic trough levels of 10–15 nmol/L [9] in obese men. Trough levels represent the therapeutic target immediately prior to the next dose and are lower than steady state targets (e.g. 13.9–24.3 nmol/L) recommended for topical treatment [10].

During weeks 1 to 8 subjects were instructed to replace all of their three principal daily meals with a VLED formulation (Optifast® VLED, Nestle, Australia) providing 640 kcal per day and two cups of low-starch vegetables. During weeks 9–10, subjects weaned their VLED and ordinary foods were gradually reintroduced. After 10 weeks, subjects had completely ceased the VLED and were instructed to follow an energy-restricted diet based on the Australian Commonwealth Scientific and Industrial Research Organisation Total Wellbeing diet (1350 kcal/d) for the remaining 46 study weeks aimed at preventing weight regain [11]. Subjects underwent weighing and individual counselling at every visit and were provided with written information to ensure dietary compliance. Subjects were advised to perform at least 30 minutes of moderate-intensity exercise each day and completed exercise questionnaires and accelerometer testing (at weeks 0, 10 and 56), with feedback given, to reinforce and encourage participation in exercise.

### Schedule of assessment and measurements

Subjects underwent long assessments at weeks 0, 10 and 56, including clinical assessment, physical function tests, accelerometer fitting (worn 7 days), questionnaires, fasting morning blood tests, dual-energy X-ray absorptiometry (DXA) scan for body composition and abdominal computed tomography (CT) scans for visceral fat, and short assessments (weeks 2, 4, 6, 16, 26, 36 and 46) for clinical assessment and to ensure dietary compliance. Adherence to the diet was estimated by measuring body weight at each study visit, with individualised feedback given.

#### Sex steroid measurements

All blood samples were drawn in the morning (8–10 am), in the fasted state. Because liquid chromatography-tandem mass spectroscopy (LCMS/MS) was not available for routine clinical use, TT was initially measured by electrochemiluminescence immunoassay used at the study hospital for routine clinical care (Roche Cobas C8000, Roche Diagnostics, Rotkreuz, Switzerland). The Austin Health intra-assay coefficient of variability (CV) was 6.9 % at 4.3 nmol/L and 5 % at 37.5 nmol/L. To confirm lowered levels, baseline TT was re-measured at study completion by validated LCMS/MS [12] from frozen baseline samples stored at –80 °C. SHBG was measured by electrochemiluminescence immunoassay (Roche Cobas C8000), Austin intra-assay CV of 3.4 % at 44 nmol/L. Free testosterone was calculated according to Vermeulen [13].

Metabolic parameters (fasting lipid profile, HbA1c, fasting glucose and c-peptide levels) and safety parameters (haemoglobin, haematocrit and prostate-specific antigen (PSA)) were measured at the study hospital with assay technology used for routine clinical care as described [14].

An independent investigator reviewed week 26 safety parameters for pre-defined withdrawal criteria: haemoglobin > 180 g/L, haematocrit > 0.54 or PSA > 5.5 μg/L.

Body composition including appendicular lean mass (ALM) corrected for height squared (ALM/height^2^) was measured by DXA (DXA Prodigy, Version 13.60; GE Lunar, Madison, WI, USA). Visceral fat was quantified from single axial CT images at the L4-5 intervertebral disc space using SliceOmatic (version 4.2; Tomovision, Montreal, Canada) by a blinded investigator (MR) with an intra-observer CV of 0.56 %. Step counts, physical and sedentary activity over 7 consecutive days were measured using the GT3x accelerometer (ActiGraph, Pensacola, FL, USA). Physical performance was assessed at weeks 0, 10 and 56 by four tests performed in duplicate and scored as the sum of the fastest times for each test in seconds: a 15 m rapid walking test, a 3 m up-and-go test, stair climbing and stair descending with a weighted vest. Handgrip was measured in the dominant hand using a hand-held medical dynamometer (Jamar 5030J1, Sammons Preston, Bolingbrook, IL, USA).

### Outcomes

The primary outcome measure was the difference in fat mass between testosterone- and placebo-treated men at study end (56 weeks) by DXA. Other main outcome measures included change in lean mass (DXA), visceral abdominal tissue (CT) and body weight. Further outcome measures included anthropometric measurements, handgrip, physical function, physical activity and metabolic parameters.

### Statistical analysis

The power analysis for this study was based on the effect of testosterone undecanoate on fat mass reduction of 5.6 kg reported in a previous RCT [15]. Given that previous studies have shown that dieting leads to loss of fat mass, we expected that the placebo group would retain some degree of fat loss by the end of the study. We therefore proposed a fat mass difference of 10 % and common standard deviation of 15.49, requiring a total of 49 subjects to achieve 90 % power for a two-sample t-test comparing mean percentage fat mass loss between the two groups. To account for a 50 % attrition rate, 100 subjects were required.

Repeated measures of main outcome continuous data were analysed using linear mixed models (LMEs) with random intercepts to account for within-individual correlation over time. LME random effect and residual normality assumptions were checked and resulted in no noteworthy violations. Restricted maximum likelihood estimates were used and the LME model covariates included factor variables ‘weeks’ and ‘treatment’ and were further adjusted for baseline testosterone and age. An intention-to-treat analysis was also carried out where the outcome measures for study dropouts were returned to baseline. Together with the LME analysis of the raw data, the LME return-to-baseline analysis provides protection against biases introduced due to missing data. Data shown are mean and 95 % confidence interval (CI). The mean adjusted difference (MAD) plus 95 % CI refers to the difference between groups of mean change in the main outcome measures over time.

Separate models with similar characteristics were used to assess other outcome data and safety variables. To compare repeated measurements of variables within groups between two time points, the t-test was used. Data shown are mean and 95 % CI. All tests were two-tailed with *P* < 0.05 denoting statistical significance. No adjustments were made for multiple comparisons on other variables. Comparison of baseline characteristics was based on the t-test or χ^2^ test in case of categorical variables. In the case of low numbers, the Fisher exact test was used. Data shown are mean (standard deviation) or median (interquartile range), based on normality testing, using the Kolmogorov-Smirnov test with Lilliefors correction. All analyses of means were complemented with Wilcoxon non-parametric tests. Similar results were found so the results were not reported. Analyses were conducted using R version 3.01 and SPSS version 22 (SPSS Inc., Chicago, IL).

The wider variation and influence of few strong responders observed in the main outcome of fat mass, typical for obesity trials, was addressed in a sensitivity analysis using a robust mixed linear model, as implemented by the r package robustlmm [16]. This model corrects for natural heteroskedasticity and the potential influence of exceptional responders by introducing a weighing algorithm and Design Adaptive Scale estimate according to Koller [16], which is less sensitive to outliers in data than the squared error loss.

## Results

### Study subjects

**Fig. 1:**
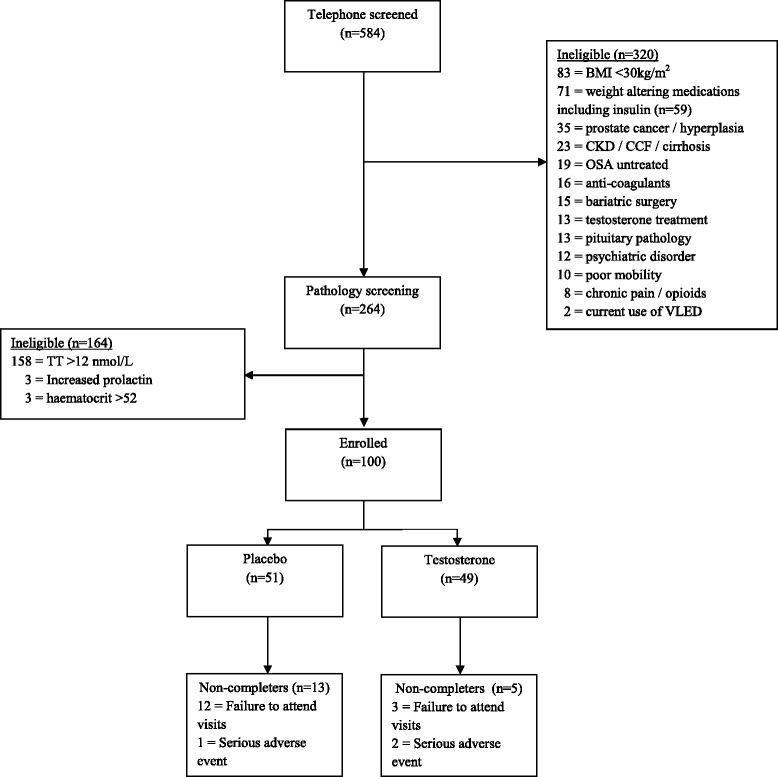
Trial profile. Shown is study enrolment and follow-up. The most common reason for non-completion was failure to attend visits. Serious adverse events are detailed in Table 4. *BMI* body mass index, *CKD* chronic kidney disease, *CCF* congestive cardiac failure, *OSA* obstructive sleep apnoea, *VLED* very low energy diet

Between April 2013 and October 2014, we assessed 584 men for eligibility. Of these, 264 men proceeded to screening investigations and 164 were ineligible, chiefly due to a TT level above 12 nmol/L (*n* = 158) (Fig. 1). The remaining 100 men were randomised to testosterone (*n* = 49, cases) or placebo (*n* = 51, controls); 82 men completed the trial, of which 44/49 (90 %) were cases and 38/51 (75 %) controls (*P* = 0.099). The most common reason for non-completion was failure to attend visits (cases = 3, controls = 12).

Baseline characteristics were comparable between the groups (Table 1). By study end, trough TT increased to 14.1 nmol/L (recommended trough range 10–15 nmol/L) in cases and 10.0 nmol/L in controls, both *P* < 0.05 compared to baseline and significantly different between groups (*P* < 0.001), with similar changes in calculated free testosterone. Luteinising hormone levels decreased from 4.5–0.4 IU/L in cases, but remained unchanged in controls (4.2–4.2 IU/L; *P* < 0.001 between groups) (Additional file 1: Figure S1).

**Table 1:** Baseline characteristics of randomly assigned study participants

	Testosterone group (*n* = 49)	Placebo group (*n* = 51)	*P* value
Age, years	54.3 (47.3–59.8)	52.8 (47.6–60.1)	0.93
Weight, kg	118.3 (15.7)	120.7 (19.6)	0.51
BMI, kg/m^2^	37.5 (34.9–40.5)	37.3 (34.7–41.6)	0.60
Waist circumference, cm	124 (118–131)	123 (117–136)	0.62
SBP, mmHg	135 (14)	130 (13)	0.06
DBP, mmHg	80 (78–88)	80 (78–85)	0.85
Handgrip, kg	44 (41–50)	46 (40–55)	0.65
Fat mass, kg	44.3 (10.0)	46.4 (10.6)	0.30
Fat mass, %	38.8 (33.5–42.3)	38.9 (36.0–43.5)	0.208
Lean mass, kg	68.1 (7.3)	67.4 (9.1)	0.67
ALM/height^2^, kg/m^2^	9.7 (1.0)	9.6 (0.8)	0.35
VAT area, mm^2^	25,088 (8617)	24,836 (9557)	0.89
Ischemic heart disease^a^	6 (12.2 %)	6 (11.8 %)	0.94
Diabetes	10 (20.4 %)	12 (23.5 %)	0.71
Metformin	3 (6.1 %)	3 (5.9 %)	0.96
Statin use	14 (28.6 %)	15 (29.4 %)	1.0
Steps per day	6378 (4761–7543)	6371 (4816–7440)	0.79
Activity (%/day)	14.2 (5.7)	13.5 (4.5)	0.49
Physical performance test (sec)	36.2 (32.5–41.6)	37.1 (33.1–40.5)	0.40
TT, nmol/L, ECLIA	8.2 (2.5)	8.4 (2.3)	0.65
TT, nmol/L, LCMS/MS	6.8 (2.0)	7.0 (1.6)	0.55
cFT, pmol/L, ECLIA	195 (58)	208 (55)	0.23
cFT, pmol/L, LCMS/MS	159 (46)	172 (44)	0.15
SHBG, nmol/L	25 (18–31)	21 (17–26)	0.17
LH, IU/L	4.5 (3.3–5.6)	4.2 (3.1–5.2)	0.70
Fasting glucose, mmol/L	5.8 (5.3–6.1)	5.8 (5.4–6.4)	0.72
HOMA-IR	3.3 (1.0)	3.6 (1.1)	0.27
HbA1c, %	6.0 (5.6–6.2)	6.1 (5.8–6.5)	0.23
Total cholesterol, mmol/L	5.1 (4.3–5.6)	4.8 (4.4–5.7)	0.60
LDL-c, mmol/L	2.9 (0.9)	2.9 (1.0)	0.99
HDL-c, mmol/L	1.1 (1.0–1.3)	1.2 (1.0–1.3)	0.58
Triglycerides, mmol/L	1.9 (1.4–2.4)	1.7 (1.2–2.5)	0.38
Haematocrit	0.43 (0.02)	0.44 (0.02)	0.003
Haemoglobin, g/L	148 (8)	152 (9)	0.014
PSA, μg/L	0.7 (0.5–1.1)	0.7 (0.5–1.2)	0.79

Data are mean (SD), median (IQR) based on normality testing, using the Kolmogorov–Smirnov test with Lilliefors correction, or number (%). *P* values were calculated for the difference between groups using *t*-test, Wilcoxon Signed Ranks Test, χ^2^ test, or Fisher exact test; *P* < 0.05 was considered significant

^a^Ischaemic heart disease denotes previous acute myocardial infarction, coronary artery bypass grafting, percutaneous transluminal coronary angioplasty

*BMI* body mass index, *DBP* diastolic blood pressure, *SBP* systolic blood pressure, *ALM* appendicular lean mass, *VAT* visceral abdominal tissue, *SHBG* sex hormone binding globulin, *TT* testosterone, *cFT* calculated free testosterone, *ECLIA* electrochemiluminescence immunoassay, *LCMS* liquid chromatography-tandem mass spectroscopy, *LH* luteinising hormone, *HbA1c* glycated haemoglobin, *LDL-c* low density lipoprotein cholesterol, *HDL-c* high density lipoprotein cholesterol, *PSA* Prostate-specific antigen

### Change in main outcome measures

At the end of the 10-week VLED phase, both cases (–12.0 kg; –14.5 to –9.5) and controls (–13.5 kg; –16.0 to –11.0) lost the same body weight, with no difference in body composition (Table 2).

**Table 2:** Change in main outcomes compared to baseline within and between groups

	Testosterone group *n* = 49	Placebo group *n* = 51	Difference between groups^a^	*P* value^b^
Fat mass, kg				
Week 0–10	–7.9 (–9.7 to –6.1)^*^	–7.5 (–9.4 to –5.7)^*^	–0.4 (–3.0 to 2.2)	0.78
Week 0–56	–9.4 (–11.3 to –7.5)^*^	–6.5 (–8.5 to –4.5)^*^	–2.9 (–5.7 to –0.2)	0.04
Fat mass, %				
Week 0–10	–3.8 (–4.9 to –2.6)^*^	–3.0 (–4.3 to –1.8)^*^	–0.7 (–2.4 to 1.0)	0.40
Week 0–56	–5.7 (–6.9 to –4.5)^*^	–2.9 (–4.3 to –1.6)^*^	–2.8 (–4.6 to –1.0)	0.003
Lean mass, kg				
Week 0–10	–3.9 (–5.3 to –2.6)^*^	–4.8 (–6.2 to –3.5)^*^	0.9 (–1.0 to 2.8)	0.36
Week 0–56	–0.6 (–2.0 to 0.8)	–4.0 (–5.5 to –2.5)^*^	3.4 (1.3 to 5.5)	0.002
VAT area, mm^2^				
Week 0–10	–7688 (–9333 to –6044)^*^	–6590 (–8267 to –4912)^*^	–1099 (–3448 to 1251)	0.36
Week 0–56	–7223 (–8921 to –5526)^*^	–4545 (–6383 to –2708)^*^	–2678 (–5180 to –176)	0.04
Body weight, kg				
Week 0–10	–12.0 (–14.5 to –9.5)^*^	–13.5 (–16.0 to –11.0)^*^	1.5 (–2.0 to 5.1)	0.40
Week 0–56	–11.4 (–13.9 to –8.8)^*^	–10.9 (–13.6 to –8.1)^*^	–0.5 (–4.3 to 3.3)	0.80

Data are mean (95 % CI)

^a^Difference between groups (mean adjusted difference) refers to the change over time across groups (linear mixed effects model)

^b^ *P* value refers to the overall significance of the change between groups during follow-up

^*^ *P* < 0.05 within group

*VAT* visceral abdominal tissue

Following resumption of normal foods as part of an energy restricted diet shown to prevent weight regain, from week 10 onwards for a further 46 weeks, body weights remained largely stable from week 10 until study end (week 56) (Additional file 1: Figure S2). In particular, men receiving testosterone maintained weight loss (*P* = 0.62), while there was weight regain in the placebo group (*P* = 0.06). At study end, cases had, compared to baseline, lost significantly more fat mass (MAD –2.9 kg (–5.7 to –0.2), *P* = 0.04), fat mass percentage (MAD –2.8 % (–4.6 to –1.0), *P* = 0.003) and visceral fat (MAD –2678 mm^2^ (–5180 to –176), *P* = 0.04), whilst regaining diet-induced loss of lean mass (MAD 3.4 kg (1.3–5.5), *P* = 0.002) (Table 2). During weeks 10–56, loss of fat mass percentage was greater in cases than in controls (MAD –2.1 % (–3.9 to –0.2), *P* = 0.03).

As the combined lean and fat mass lost in controls was similar to the amount of fat mass lost in the cases, the difference in body weight change at study end was no different between groups (MAD –0.5 kg (–4.3 to 3.3), *P* = 0.80) (Table 2). Age, baseline TT, luteinising hormone and SHBG levels were all not predictive of changes in body composition after 56 weeks in the trial. Further, baseline fat mass did not interact with the changes in body composition. In addition, adjustment for physical activity did not alter the findings.

### Other outcomes

Compared to controls, cases retained higher ALM/height^2^ (0.45, *P* < 0.001) (Table 3). Cases had a significant increase in handgrip strength compared to placebo (3.6 kg, *P* = 0.03). Both groups increased their daily step counts (*P* < 0.01) and activity levels (*P* < 0.05) at 10 weeks. At study end, daily step count was increased significantly in cases by 931 steps (*P* = 0.03), as was percentage of daily non-sedentary time (+1.5 %, *P* = 0.03). This was due to spending less time in sedentary and more time in light activity (*P* = 0.016). No significant changes in either outcome were observed in controls nor between groups at study end (Table 3). Both groups improved on physical performance testing but there was no difference between groups at study end. Similarly, both groups had improvements in metabolic parameters without between-group differences (Table 3).

**Table 3:** Other outcomes, change in outcome at study end from baseline

	Testosterone group *n* = 49	Placebo group *n* = 51	Between group	*P* value^a^
BMI, kg/m^2^	–3.7^*^ (–4.7 to –2.7)	–3.6^*^ (–4.8 to –2.3)	–0.1 (–1.7 to 1.4)	0.85
Waist circumference, cm	–11^*^ (–14 to –8)	–8^*^ (–12 to –5)	–3 (–7 to 2)	0.21
SBP, mmHg	–1 (–6 to 3)	–1 (–6 to 3)	0 (–6 to 6)	0.94
DBP, mmHg	0 (–3 to 3)	–1 (–4 to 2)	1 (–3 to 5)	0.62
Handgrip, kg	1.7 (–0.5 to 3.8)	–1.9 (–4.3 to 0.5)	3.6 (0.4 to 6.7)	0.03
ALM/height^2^, kg/m^2^	0.12 (–0.02 to 0.26)	–0.33^*^ (–0.50 to –0.15)	0.45 (0.22 to 0.67)	<0.001
Step count per day	931^*^ (116 to 1746)	606 (–186 to 1399)	325 (–794 to 1443)	0.56
Activity, %/day	1.5^*^ (0.1 to 2.9)	0.7 (–0.6 to 2.1)	0.76 (–1.2 to 2.7)	0.43
Physical function test, sec	–3.1^*^ (–4.8 to –1.5)	–2.7^*^ (–4.1 to –1.3)	–0.4 (–2.6 to 1.7)	0.67
TT, nmol/L, ECLIA	7.4^*^ (5.7 to 9.1)	1.8^*^ (0.4 to 3.1)	5.5 (3.4 to 7.7)	<0.001
cFT, pmol/L, ECLIA	49^*^ (37 to 60)	4 (–3 to 10)	46 (32 to 59)	<0.001
SHBG, nmol/L	4^*^ (2 to 6)	7^*^ (3 to 10)	–3 (–6.4 to 1.1)	0.16
LH, IU/L	–4.0^*^ (–4.7 to –3.4)	0.2 (–0.5 to 1.0)	–4.3 (–5.2 to –3.3)	<0.001
Fasting glucose, mmol/L	–0.5^*^ (–0.8 to –0.25)	–0.3^*^ (–0.5 to 0.0)	–0.3 (–0.6 to 0.1)	0.19
HOMA-IR	–0.8^*^ (–1.1 to –0.5)	–0.6^*^ (–0.9 to –0.2)	–0.2 (–0.7 to 0.3)	0.38
HbA1c, %	–0.5^*^ (–0.6 to –0.3)	–0.3^*^ (–0.5 to –0.2)	–0.1 (–0.3 to 0.1)	0.19
Cholesterol, mmol/L	–0.18 (–0.4 to 0.0)	0.0 (–0.2 to 0.2)	–0.19 (–0.5 to 0.1)	0.23
LDL-C, mmol/L	–0.1 (–0.3 to 0.1)	–0.1 (–0.3 to 0.2)	0.0 (–0.3 to 0.3)	0.88
HDL-C, mmol/L	0.1^*^ (0.1 to 0.2)	0.1^*^ (0.0 to 0.2)	0.0 (–0.1 to 0.1)	0.66
Triglycerides, mmol/L	–0.6^*^ (–0.9 to –0.4)	–0.4^*^ (–0.6 to –0.1)	–0.3 (–0.6 to 0.1)	0.17
Haematocrit	0.04^*^ (0.03 to 0.05)	–0.005^*^ (–0.009 to –0.001)	0.04 (0.03 to 0.05)	<0.001
Haemoglobin, g/L	14^*^ (11 to 17)	–2^*^ (–3 to 0)	15 (12 to 19)	<0.001
PSA, μg/L	0.3^*^ (0.2 to 0.5)	0.1^*^ (0.0 to 0.3)	0.2 (0.0 to 0.4)	0.10

Shown are within- and between-group differences between baseline and study end

The data are mean (95 % CI).

^a^ *P* value refers to difference between groups at study end

^*^ *P* < 0.05 versus baseline within group

*BMI* body mass index, *DBP* diastolic blood pressure, *SBP* systolic blood pressure, *ALM* appendicular lean mass, *VAT* visceral abdominal tissue, *SHBG* sex hormone binding globulin, *TT* testosterone, *cFT* calculated free testosterone, *ECLIA* electrochemiluminescence immunoassay, *LCMS* liquid chromatography-tandem mass spectroscopy, *LH* luteinising hormone, *HbA1c* glycated haemoglobin, *LDL-c* low density lipoprotein cholesterol, *HDL-c* high density lipoprotein cholesterol, *PSA* Prostate-specific antigen

### Sensitivity analyses

Outcomes were unchanged after imputation of missing values using an intention-to-treat analysis and return-to-baseline for missing data (Additional file 1: Table S1; MAD for fat mass (–3.3 kg, *P* = 0.014), visceral abdominal tissue (–3223 mm^2^, *P* = 0.007) and for lean mass (2.3 kg, *P* = 0.015)). Similar findings were also found using non-parametric tests.

In an additional sensitivity analysis, when re-analysed with a robust linear mixed model (see Methods), the main outcome fat loss after 56 weeks was more pronounced in the testosterone group, compared to the placebo group (–4.1 kg (–5.6 to –2.7), *P* < 0.01).

Compared to participants completing the study (*n* = 81), non-completers (*n* = 19) lost less body weight (–8.2 kg (–12.1 to –6.2) vs. –13.4 (–17.2 to –9.9), *P* = 0.027), and less fat mass (–5.2 kg (–7.2 to –3.7) vs. –8.1 (–10.7 to –4.9), *P* = 0.009) at week 10, the end of the VLED phase. Similar findings were also observed if non-completers in the placebo group were analysed separately (data not shown).

### Adverse events

There was no between-group difference in overall adverse events, or serious adverse events which were few (Table 4). One case had a rise in haemoglobin above the predetermined safety limit of above 180 g/L occurring at study end. Three men were withdrawn from the study due to PSA rise (cases = 1) and major cardiovascular events (cases = 1, controls = 1).

**Table 4:** Incidence of adverse events

	Testosterone group *n* = 49	Placebo group *n* = 51
Overall		
Any adverse event	10	8
Withdrawal due to serious adverse event^a^	2	1
Prostate		
Rise in prostate-specific antigen by > 1.0 μg/L	5^*^	0
Prostatitis	1	0
Rise in haemoglobin to > 180 g/L^b^	1	0
Cardiovascular	1	1
Gastrointestinal	0	2
Dermatological	2	4
Neurological	0	1

^*^ *P* = 0.02

^a^A total of three men were withdrawn from the study: one man receiving testosterone experienced angina requiring coronary artery stent insertion, one man receiving testosterone had a rise in prostate-specific antigen above the pre-determined safety level (>5.5 μg/L) at week 26, one man receiving placebo experienced ventricular fibrillation and was resuscitated

^b^The rise in haemoglobin above the pre-determined safety level (>180 g/L) occurred at study end

## Discussion

The major novel findings of this RCT are that, among obese men with low to low-normal testosterone submitted to a weight loss program, testosterone treatment decreased total fat mass and visceral adipose tissue, and protected against loss of total and appendicular lean mass. At the end of the initial 10-week VLED phase, while men lost substantial amounts of weight similar to previous successful VLED studies [11], there were no differences in weight loss or body composition changes between the two groups. However, differences emerged in the weight maintenance phase, during which men receiving testosterone maintained weight loss (*P* = 0.62), while there was marginal weight regain in the placebo group (*P* = 0.06). At study end, there were marked differences in body composition between groups, and men receiving testosterone had greater reductions of fat mass (–2.9 kg) and visceral fat area (–2678 mm^2^) compared to placebo. After the VLED phase, men receiving testosterone regained lean mass (3.3 kg, *P* < 0.001) in contrast to placebo (0.8 kg, *P* = 0.29), so that at study end, lean mass was 3.4 kg higher in testosterone-treated men. Overall, our results indicate that, compared to men receiving placebo who lose both fat and muscle mass during diet, testosterone treatment shifts this weight loss to almost exclusive fat mass loss.

Our trial has several strengths distinguishing it from previous testosterone trials, most importantly, the successful implementation of a rigorous weight loss program and the exclusive focus on men with established obesity. By contrast, previous RCTs examining the effects of testosterone on body composition recently meta-analysed [8] were neither designed for weight loss nor had obesity as a selection criterion. Moreover, only a few studies, not all placebo controlled, have combined testosterone treatment with lifestyle measures. A recent meta-analysis of these studies [17] suggested that testosterone treatment may have added benefits on body composition, consistent with our findings. We confirmed lowered baseline testosterone levels using LCMS/MS technology [12], used intramuscular testosterone eliminating compliance issues for a relatively long duration in a double-blind placebo controlled design, and attrition rate was relatively low. Compared to men completing the study, non-completers had lost less body weight and less fat mass at the end of the VLED phase of the study. Therefore, if anything, this would be expected to underestimate the benefits of testosterone treatment, especially as the drop-out rate was higher among men assigned to placebo compared to testosterone treatment.

Although it may be expected that the effects of testosterone treatment are attenuated in the context of a rigorous weight loss program, the reduction of fat mass observed here compares favourably with the 1.6–2.0 kg reduction reported in meta-analyses of RCTs not incorporating weight loss measures [18, 19]. This may be because we focused on obese men with a confirmed low testosterone receiving effective testosterone treatment. This may also explain the robust increase in lean mass of 3.4 kg, compared to 1.6–2.7 kg in previous meta-analyses [18, 19]. Testosterone treatment did not prevent the loss of lean mass during the 10-week VLED suggesting that testosterone treatment lacks anabolic actions during acute severe caloric restriction. However, 10 weeks of treatment may be too short to manifest changes in body composition, since testosterone-mediated changes in lean mass are evident only after several months [18, 19].

Testosterone treatment significantly reduced the metabolically important visceral fat even in the context of a weight loss program. Previous RCTs of testosterone therapy, while not incorporating a weight loss program, did not find a consistent reduction in visceral fat [14, 15, 20, 21], most likely because of small trial size [15], use of oral testosterone therapy [20], or less precise methodology to quantify visceral adipose tissue [20]. None of these RCTs specifically targeted obese men.

Interestingly, the differences in body composition were evident despite the modest increase in endogenous testosterone levels in placebo-treated men similar to previous weight loss studies [22]. Indeed, this increase by 2.9 nmol/L in TT and 30.3 pmol/L in free testosterone with 10.8 % weight loss was very similar to that reported in a meta-analysis of low caloric diet studies [23]. Thus, the endogenous rise in testosterone subsequent to diet appears not to be sufficient to prevent diet-associated loss of lean mass.

What are the potential mechanisms by which testosterone treatment leads to these changes in body composition? Testosterone, via androgen receptor signalling, inhibits stem cell differentiation into adipocytes and favours myogenesis [6]. Androgen receptor signalling in mature adipocytes promotes lipolysis [24] and activates anabolic pathways in myocytes [25]. The effect on fat mass may also be mediated by aromatisation to estradiol [26]. Testosterone may also have motivational effects leading to increased physical activity; in RCTs, testosterone treatment reduces fatigue and inertia [27], and androgen-deficient mice have decreased voluntary activity [28]. We advised subjects to perform at least 30-minutes of moderate-intensity exercise each day. Subjects completed exercise questionnaires and accelerometer testing, with feedback given, to reinforce and encourage participation in exercise. Both men receiving testosterone and placebo increased their activity during the weight loss phase. However, only men receiving testosterone (*P* = 0.03) but not placebo (*P* = 0.28) maintained increased activity levels at study end, suggesting that increased physical activity may have contributed to the observed changes in body composition in testosterone-treated men.

Supervised exercise programs may promote loss of fat mass and attenuate loss of muscle mass during weight loss, but are less effective than caloric restriction to achieve weight loss. Exercise interventions are not well characterised for obese men with low testosterone and require high volume interventions, which may be difficult to achieve even in a dedicated RCT [29]. Only few studies have randomised obese men receiving caloric restriction to exercise programs. The effects of testosterone reported here compare favourably; systematic reviews have estimated that the addition of exercise to energy restriction increases the loss of fat mass by 1.6 kg [30] but does not fully protect against the loss of lean mass that occurs with diet, reducing this by 50 % [2]. In the Look Ahead study, men, despite assignment to an intensive lifestyle intervention, lost 2.5 kg of lean mass in the first study year [31].

Metabolic parameters, evidenced by decreases in HOMA-IR, HbA1c, triglycerides, and increases in HDL levels improved in both groups. Testosterone treatment had no added benefit, despite resulting in changes in body composition expected to be metabolically favourable. Our study was not designed to examine this outcome, and men enrolled were relatively healthy, with a low proportion of men being diabetic or dyslipidaemic at baseline.

Consistent with previous studies, we observed a significant increase in haematocrit in testosterone-treated men. Overall, serious adverse events were few and not statistically different between groups, although this study was not powered to assess safety. Ten percent of the participants assigned to testosterone treatment had an increase of above 1 μg/L in PSA during the study, similar to the 6 % among men allocated to testosterone in the recent testosterone trials [32]. However, the significance of this biochemical increase is uncertain, and although definitive long-term studies are lacking, the current evidence does not suggest that testosterone treatment leads to clinically meaningful adverse prostate outcomes.

Limitations include the enrolment of relatively healthy men motivated to lose weight subjected to a professionally administered diet and frequent monitoring. Despite preservation of lean mass, testosterone treatment, with the exception of increased grip strength, did not affect muscular performance. Previous studies have suggested that testosterone treatment improves physical performance primarily in frail, mobility-limited men [33, 34]. Although we did not include a supervised exercise program, exercise recommendations were reinforced at every visit, and men assigned to testosterone but not placebo, had increased activity levels. We selected participants based on a TT of less than 12 nmol/L to include men with modestly reduced levels typical of the majority of obese men [3]. This allowed us to capture the large population in whom testosterone treatment (be it replacement or pharmacological) is more controversial than in men with more profound reductions in testosterone, or indeed with organic hypogonadism. While TT may reflect adaptation to obesity-associated lowering of SHBG, it is important to emphasize that 97 % of our study population had a baseline free testosterone (calculated from LCMS/MS total testosterone) of less than 243 pmol/L, the lower limit reported for healthy young men [4], and 89 % a level of less than 220 pmol/L, the cut-off for late onset hypogonadism [35]. While we did not find an added effect of testosterone treatment on diet-induced loss of body weight in this 56-week study, it is possible that the duration of our study was insufficient, given that a recent meta-analysis of observational studies has suggested that testosterone treatment may be associated with time-dependent weight loss that may only be evident after 2 years of treatment [36]. Finally, our study was not designed to examine cardio-metabolic outcomes.

## Conclusions

Among obese men with a low to low-normal testosterone typical for the majority of obese men, testosterone treatment augmented diet-induced loss of total fat and visceral fat mass, and preserved lean mass so that, in contrast to placebo-treated men who lost both lean and fat mass, diet-induced weight loss during testosterone treatment was almost exclusively due to the loss of body fat.

## Additional file

Additional file 1: Table S1. Change in main outcomes compared to baseline within and between groups analysed by intention to treat. **Figure S1.** Circulating testosterone and luteinising hormone levels in placebo- and testosterone-treated men. Shown are (median, IQR) circulating total testosterone (TT) (A), calculated free testosterone (B) and luteinising hormone (C) levels at the indicated time points during the trial in men receiving placebo or testosterone, respectively. Circulating testosterone levels at 26 and 56 weeks represent trough levels, which were in the therapeutic trough range (10–15 nmol/L for TT) in testosterone-treated men. By contrast, testosterone levels at week 10 were obtained 4 weeks after injection of the study drug and represent a 4-week post injection peak, which is higher than the recommended trough range. **Figure S2.** Body weights in placebo and testosterone treated men. Shown are mean (95 % CI) body weights in kilogram in placebo- (grey line) and testosterone-treated men from baseline to 56 weeks. (PDF 321 kb)

## Abbreviations

ALM: appendicular lean mass
BMI: body mass index
cFT: calculated free testosterone
CI: confidence interval
CT: computed tomography
CV: coefficient of variability
DXA: dual-energy X-ray absorptiometry
LCMS/MS: liquid chromatography-tandem mass spectroscopy
LME: linear mixed models
MAD: mean adjusted between group difference
PSA: prostate-specific antigen
RCT: randomized, double-blind, placebo-controlled trial
SHBG: sex hormone binding globulin
TT: total testosterone
VLED: very low energy diet
