# Supplementary material for: Effects of testosterone treatment on body fat and lean mass in obese men on a hypocaloric diet: a randomised controlled trial
Source: BMC Med. 2016 Oct 7;14:153. doi: 10.1186/s12916-016-0700-9 (PMC5054608; doi:10.1186/s12916-016-0700-9)
Supplement: Additional file 1: Table S1. — Change in main outcomes compared to baseline within and between groups analysed by intention to treat. Figure S1. Circulating testosterone and luteinising hormone levels in placebo- and testosterone-treated men. Shown are (median, IQR) circulating total testosterone (TT) (A), calculated free testosterone (B) and luteinising hormone (C) levels at the indicated time points during the trial in men receiving placebo or testosterone, respectively. Circulating testosterone levels at 26 and 56 weeks represent trough levels, which were in the therapeutic trough range (10–15 nmol/L for TT) in testosterone-treated men. By contrast, testosterone levels at week 10 were obtained 4 weeks after injection of the study drug and represent a 4-week post injection peak, which is higher than the recommended trough range. Figure S2. Body weights in placebo and testosterone treated men. Shown are mean (95 % CI) body weights in kilogram in placebo- (grey line) and testosterone-treated men from baseline to 56 weeks. (PDF 321 kb) [file 12916_2016_700_MOESM1_ESM.pdf]

Table S1: Change in main outcomes compared to baseline within and between groups analysed by ITT

	Testosterone group N=49	Placebo group N=51	Difference between groups^	p-value#
**Fat mass (kg)**				
week 0-10	-7.7 (-9.5, -5.9)*	-7.2 (-9.0, -5.4)*	-0.5 (-3.1, 2.1)	0.71
week 0-56	-8.3 (-10.1, -6.4)*	-5.0 (-6.9, -3.2)*	-3.3 (-5.8, -0.7)	0.014
**Fat mass (%)**				
week 0-10	-3.7 (-4.8, -2.5)*	-3.0 (-4.1, -1.8)*	-0.7 (-2.4, 0.9)	0.39
week 0-56	-5.0 (-6.1, -3.8)*	-2.4 (-3.5, -1.2)*	-2.6 (-4.3, -1.0)	0.002
**Lean mass (kg)**				
week 0-10	-3.9 (-5.2, -2.5) *	-4.6 (-5.9, -3.3) *	0.7 (-1.1, 2.6)	0.44
week 0-56	-0.7 (-2.0, 0.7)	-3.0 (-4.3, -1.6) *	2.3 (0.5, 4.2)	0.015
**VAT area (mm^2)**				
week 0-10	-7,581 (-9,209, -5,934) *	-6,247 (-7,892, -4,602) *	-1,334 (-3,648, 980)	0.26
week 0-56	-6,572 (-8,200, -4,944)*	-3,349 (-4,994, -1,705) *	-3,223 (-5,537, -909)	0.007
**Body weight (kg)**				
week 0-10	-11.7 (-14.2, -9.2) *	-12.9 (-15.4, -10.4) *	1.2 (-2.4, 4.7)	0.51
week 0-56	-10.4 (-12.9, -7.9)*	-8.2 (-10.7, -5.7) *	-2.2 (-5.7, 1.4)	0.23

Data are mean (95% CI)

^Difference between groups (mean adjusted difference) refers to the change over time across groups (linear mixed effects model)

#the p-value refers to the overall significance of the change between groups during follow-up

*p <0.05 within group

Figure S1. Circulating testosterone and luteinising hormone levels in placebo and testosterone treated men.

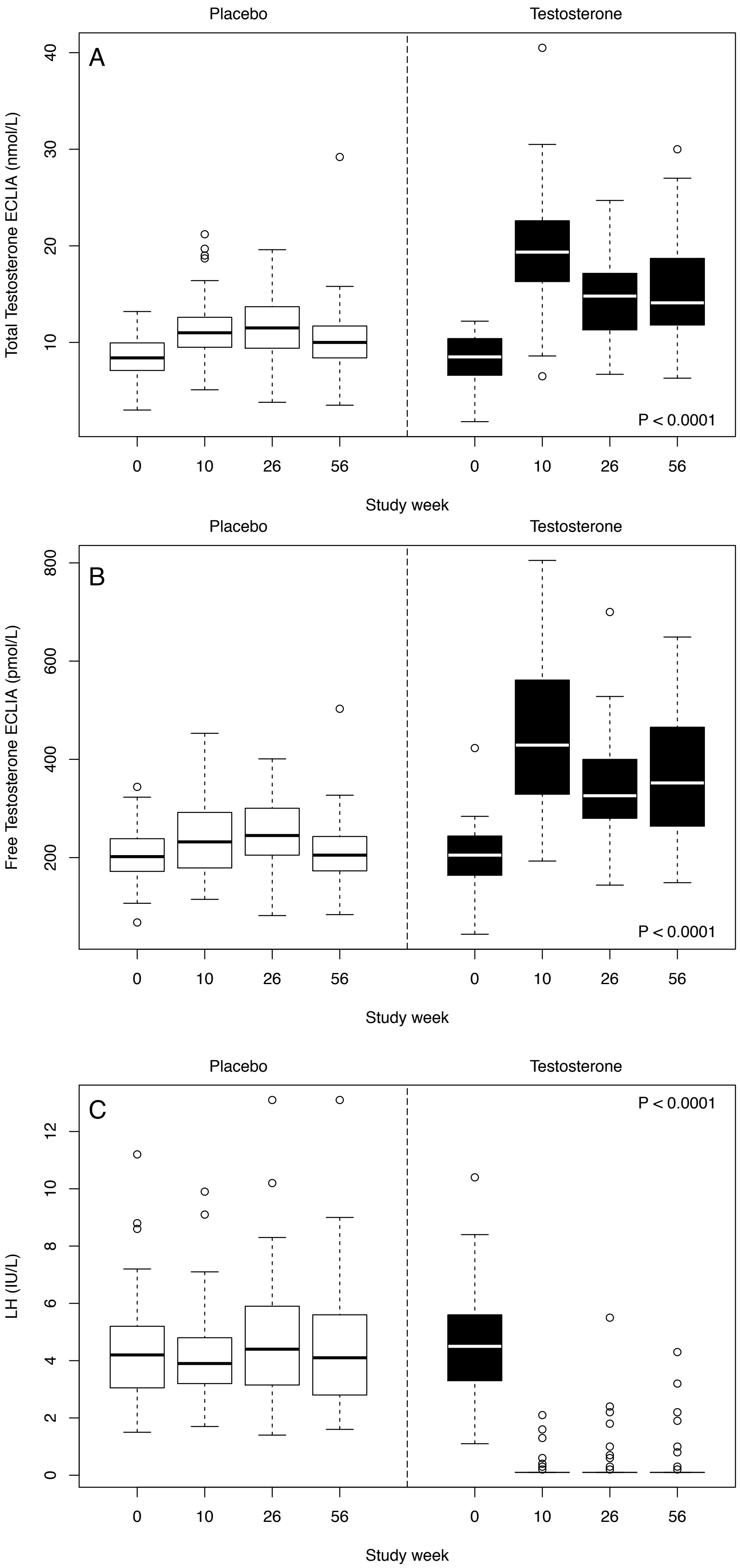

Figure S2. Body weights in placebo and testosterone treated men.

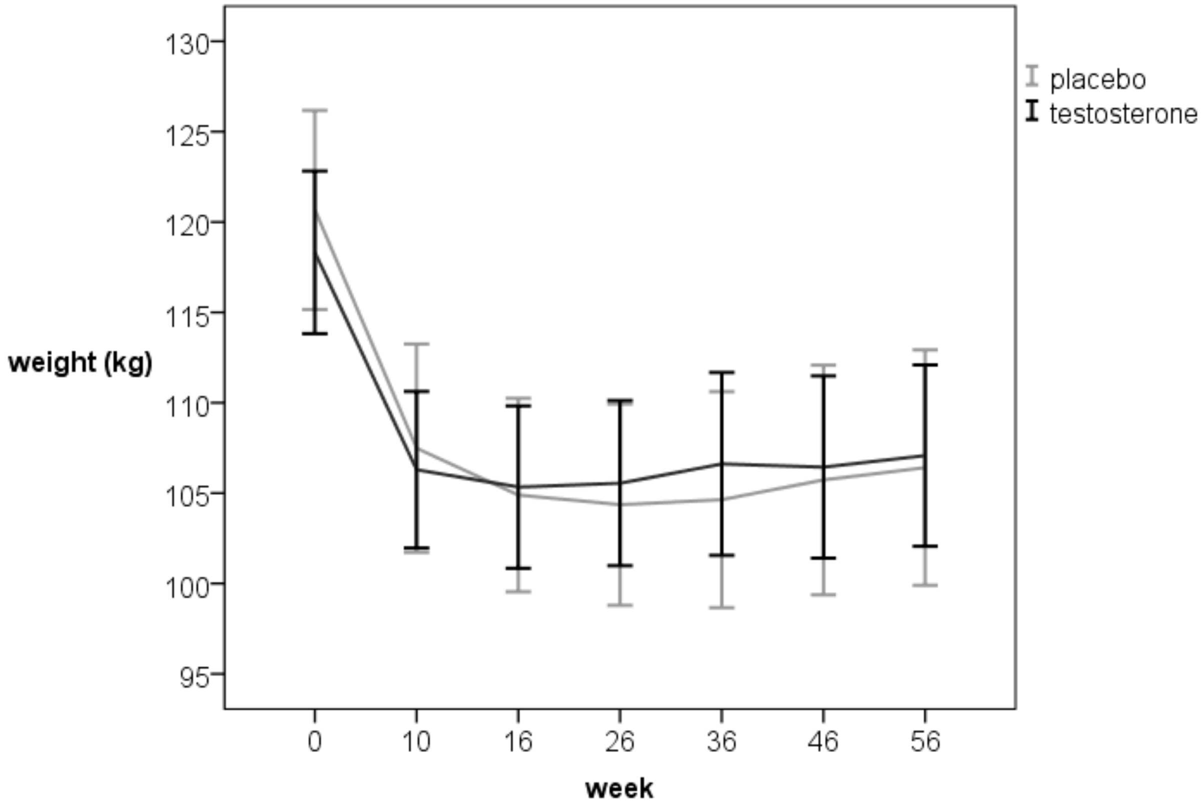

testosterone (n)	49	48	47	47	44	44	44
placebo (n)	51	49	46	43	39	38	38
